# Clinical and Serological Characteristics of a Monocentric Cohort of Patients Affected by Interstitial Pneumonia with Autoimmune Features (IPAF)

**DOI:** 10.31138/mjr.34.2.180

**Published:** 2023-06-30

**Authors:** Claudia Canofari, Andrea Vendola, Annamaria Iuliano, Loreta Di Michele, Alfredo Sebastiani, Andreina Gubbiotti, Gian Domenico Sebastiani

**Affiliations:** 1 Rheumatology Unit, Az. Osp. San Camillo-Forlanini Hospital Rome, Italy,; 2 Rheumatology, Allergology and Clinical Immunology, Department of Systems Medicine, University of Rome Tor Vergata, Rome, Italy,; 3 Pulmonary Interstitial Diseases Unit, UOSD interstiziopatie polmonari, Az Osp. San Camillo-Forlanini Hospital, Rome, Italy,; 4 Clinical Pathology Unit, Az. Osp. San Camillo-Forlanini Hospital, Rome, Italy

**Keywords:** IPAF, classification criteria, follow-up

## Abstract

**Introduction:**

Interstitial lung diseases (ILDs) are diseases characterised by excessive deposition of collagen matrices in the pulmonary interstitium. Some of them are considered idiopathic (idiopathic pulmonary fibrosis – IPF), others are related to known pathologies such as connective tissue diseases (CTDs-ILD). Patient affected by ILD and features referable to CTD, not satisfying CTD criteria, are called Interstitial pneumonia with autoimmune features (IPAF) patients.

**Objective:**

The aim of this report was to investigate clinical and serologic features of a monocentric cohort of patients with IPAF. Another objective was to describe the autoantibody profile, clinical features, High Resolution Computerised Tomography (HRCT) and Nailfold Video Capillaroscopy (NVC) patterns.

**Methods:**

36 IPAF patients were consecutively enrolled. Clinical, serological, and morphological features were collected.

**Results:**

36 consecutive IPAF patients were enrolled from January 2021 to January 2022. Raynaud’s phenomenon was the most frequent symptom identified. We also described other signs and symptoms not included in IPAF criteria. 36,1% of patients demonstrated a Usual Interstitial Pneumonia (UIP) pattern by HRCT. Pulmonary arterial pressure estimation (PAPs) resulted elevated (≥ 25 mmHg) in 6 patients. Antinuclear antibodies (ANA) ≥ 1/80 was the most frequent autoantibody, followed by anti-Ro, in patients with UIP pattern and Non-Specific Interstitial Pneumonia (NSIP) pattern at HRCT. NVC highlighted non-specific microangiopathy as the most common pattern especially in UIP patients.

**Conclusions:**

This paper may contribute to stimulate the interest in better characterisation of clinical, serologic, and instrumental features for IPAF patients by redefining IPAF classification criteria in order to treat them as best as possible.

## INTRODUCTION

Interstitial lung diseases (ILDs) are a large group of diseases characterized by excessive deposition of collagen matrices in the pulmonary interstitium. Some of them are considered idiopathic (idiopathic pulmonary fibrosis – IPF), others are related to smoking, environmental or occupational exposure, while some others are associated to a connective tissue disease (CTD-ILD), like Systemic Lupus Erythematosus, Rheumatoid Arthritis, Systemic Sclerosis, Sjögren Syndrome, etc.^1^

A growing number of studies have underlined the presence of a subset of patients with interstitial lung disease and at least one clinical/serological feature referred to autoimmune disorder, not fulfilling specific criteria for connective tissue diseases (CTD). In 2015, a task force by the European Respiratory Society (ERS) and American Thoracic Society (ATS) proposed to define this condition as Interstitial Pneumonia with Autoimmune Features (IPAF).^2^

According to the current research criteria patients should have an idiopathic interstitial pneumonia and at least one feature from at least two of clinical, serologic, morphologic domains to be classified as IPAF. However, these criteria show some limits: they have not been validated and some considerations are in research agenda also. The current IPAF criteria rely much on serologies. Especially in case of Non-Specific Interstitial Pneumonia (NSIP) or Organising Pneumonia (OP), the presence of a qualifying serology is sufficient to classify the patient as IPAF.

However, should be noted that serologic testing is highly variable. ELISA based assays are very performing in identifying extractable nuclear antigens such as Sm, dsDNA Ro/SSA, La/SSB, etc. but they are unable to assess the presence of non-specific anti-nuclear antibodies – even when present at high titre. The inclusion of myositis specific and associated antibodies (eg, antisynthetase, Pm-Scl, and MDA5) is another point of reflection, because these patients may not truly be undifferentiated.

The classification fails to capture some features also, for example the Usual Interstitial Pneumonia (UIP) pattern at High Resolution Computerized Tomography (HRCT) and many clinical features that may underline a connective tissue disease. Thus, data from retrospective and prospective studies may help to redefine IPAF classification criteria based on collected evidence.

The aim of this observational study was to investigate the clinical and serologic features of a monocentric cohort of patients with IPAF. Another objective was to describe the autoantibody profile, clinical features, HRCT and Nailfold Videocapillaroscopy (NVC) patterns.

## METHODS

The study was performed according to the declaration of Helsinki, approved by the local ethics committee and all patients signed an informed consent.^3^

Study was conducted at the ILD clinic of the Rheumatology Unit of San Camillo–Forlanini Hospital in Rome.

Patients affected by ILD with at least one clinical/serological feature of autoimmune disorder according to Fischer criteria,^2^ were consecutively enrolled. Patients affected by ILD from other causes were excluded.

The following data were collected for each patient:

- demographic findings (sex, ethnicity, age at the disease onset, age at the diagnosis, and diagnostic delay)
- comorbidities (diabetes mellitus, hypertension, dyslipidaemia, osteoporosis)
- body Mass Index (BMI)
- smoking habit
- environmental exposure
- modality of onset (acute/chronic) of the first respiratory symptom (cough and dyspnoea after mild/moderate efforts)
- the presence of possible underlying CTDs (presence / absence of arthralgia, polymyalgia symptoms, spreading myalgias, Raynaud’s phenomenon, fever, dry eyes and mouth, dysphagia; patient’s signs such as arthritis, mechanic hands, Gottron sign and papules, heliotrope rash, distal ulcers, V sign, calcinosis, puffy hands, telangiectasias, and laboratory alterations such as elevation of inflammatory markers and muscle enzymes).

NVC was performed using VideoCap 3.0. Four fingers of each hand were analysed (thumbs were excluded). For each finger, two images (from right and from left side of median line) were captured, stored, and analysed later.

For each capillaroscopic image, the following parameters were evaluated: linear capillary density (number of capillaries/mm), presence of giant capillaries, tortuosity, avascular area, capillary disarrangement, and neo-angiogenic features (number of bushy and strangely shaped capillaries/mm).

Four different patterns were distinguished:

- normal pattern;
- non-specific pattern, characterised by 7–10 capillaries/mm, less than 50% tortuous loops, arranged in parallel rows, with no haemorrhages or neoangiogenesis features;
- non-specific microangiopathy characterized by decreased capillary density, more than 50% tortuous loops, enlarged and/or disarranged loops, more than 50% neoangiogenesis, and/or microhemorrogia.^4^
- scleroderma pattern (early, active, late) as defined by Cutolo et al.^5^

Each patient underwent a visit with a trained pneumologists, pulmonary function tests (PFTs) with diffusing capacity of the lungs for carbon monoxide (DLCO).

DLCO and the value of Forced Vital Capacity (FVC) were reported as percentages of the predicted value of each parameter and corrected for age, gender, and height.

The value of FVC <80% and DLCO < 60% of predicted value were considered abnormal respectively. Furthermore, a HRCT was performed in every patient, and all images were scored by a thoracic radiologist experienced in ILD.

According to HRCT, patients were classified in different patterns: UIP, cellular Non-Specific Interstitial Pneumonia (cNSIP), fibrotic Non Specific Interstitial pneumonia (fNSIP), Lymphocytic Interstitial Pneumonia (LIP), OP, others.^6^

Additional investigations were performed such as echocardiography with mean pulmonary artery pressure estimation (PAPs), electromyography (EMG), and muscle magnetic resonance imaging when clinically indicated. The administration of drugs was recorded for each patient with relative dosage, including corticosteroids, conventional disease-modifying antirheumatic drugs (cDMARDs), biological disease-modifying antirheumatic drugs (bDMARDs), and antifibrotic agents.

Laboratory tests performed are shown in **Table 1**; antinuclear antibodies (ANA) were analysed with indirect immunofluorescence assay (IIFA) on HEp-2 cells; anti-double stranded DNA antibodies (anti-dsDNA) were analysed with chemiluminescence technique (CLIA), then confirmed with IIFA on Crithidia luciliae. CLIA method was used for extractable nuclear antigen antibodies (ENA) screening. The positivity was confirmed with immunoblotting analysis (**Table 1**).

**Table 1.** Laboratory tests.

**Spectrophotometry method**	Complete blood count (CBC)
	Urinalysis
	Serum protein electrophoresis (SPEP)
	Serum creatinine (Cr)
	Serum Glutamic Pyruvic Transaminase (SGPT)
	Lactic dehydrogenase (LDH)
	Serum Glutamic Oxaloacetic Transaminase (SGOT)
	Creatine phosphokinase (CPK)
**Wintrobe method**	Erythrocyte sedimentation rate (ESR)
**Nephelometric technology**	C-reactive protein (CRP) Complement components (C3, C4) Rheumatoid Factor (RF)
**CLIA method**	ACPA IgG/IgM, ACA IgG/IgM, beta2GPI antibodies IgG/IgM
**CLIA method CTD (ENA screening)**	Sm, RNP, SSA/Ro52kD, SSA/Ro60kd, SSB, Scl70, Jo1, Mi-2, PCNA, Ku, Th/To, PMScl, dsDNA, RNA -Polymerase III, Ribo-P,CENP-B
**Immunoblotting ENA Profile 19AG**	Nucleosomes, dsDNA, Istoni, Sm, RNP, Sm/RNP, SSA/Ro60kd, SSA/Ro52kd, SS-B, Scl70, RNApol III, Ku, PM-Scl 100, Mi-2, Jo1, CENP-A/B, PCNA, DFS-70, Ribo-P
**Immunoblotting Scleroderma**	Scl-70, CENP-A, CENP-B, PM-Scl100, PM-Scl75, Ku, RNA polymerase III, RNP68kD/A/C, Th/To, Fibrillarina, NOR-90, SSA/Ro52kD
**Immunoblotting Myositis Profile 12 antigens IgG DOT**	Jo-1, PL-7, PL-12, EJ, SRP, Mi-2, MDA-5, TIF1-y, SSA/Ro52kD, SSA/Ro60 kD, SAE1,SAE2 and NXP-2 .

ACPA: anti–citrullinated peptide antibodies, ACA: anti-cardiolipin antibodies, beta2 GPI: anti-beta2-glycoprotein I, CLIA: chemiluminescence-immunoassay, ENA: Extractable Nuclear Antigen.

All data were collected in an electronic database and analysed with statistical software GraphPad Prism 7.0.

## RESULTS

In this study, 36 consecutive patients with a diagnosis of IPAF were enrolled, from January 2021 to January 2022, aged ≥ 18 yrs: 25 females (69,4%) and 11 males (30,6%). Caucasian patients composed 91,7% of the cohort, followed by African American patients (5,5%) and Asian patients (2,8%). Among them, the mean age at disease onset was 64,1 ± 9,3 years, the mean age at diagnosis was 66,3 ± 9,3 years with a mean diagnostic delay of 20,2 ± 27,2 months and an average duration of disease of 32,4 ± 34 months (Tab.2).

**Table 2.** Demographic and clinical characteristics. Data are presented as mean ± SD or n (%).

**Age**	
At disease onset (yrs)	64,1 ± 9,3
At diagnosis (yrs)	66,3 ± 9,3
Diagnostic delay (months)	20,2 ± 27,2

**Sex**	
Female	25 (69,4)

**Race/ethnicity**	
Caucasian	33 (91,7)
Afro-american	2 (5,5)
Asian	1 (2,8)

**Smoker**	
actual or past	8 (22,2)

**Raynaud phenomenon**	9 (25)

**Nailfold capillaroscopy pattern**	
Normal	1 (2,8)
Non-specific	14 (38,9)
Non-specific microangiopathy	18 (50)
Early scleroderma pattern	0
Active scleroderma pattern	3 (8,3)
Late scleroderma pattern	0

**Puffy hands**	4 (11,1)

**Telangiectasias**	2 (5,5)

**BP >150/90 or treatment with antihypertensive drugs**	15 (41,7)

**PAPs > 25 mmHg**	7 (19,4)

**Dysphagia**	2 (5,5)

**Fever**	5 (13,9)

**Arthritis**	6 (16,7)

**Spreading myalgias**	1 (2,8)

**Dry eye**	3 (8,3)

**Dry mouth**	4 (11,1)

**First respiratory symptom**	
Cough	16 (44,4)
Dyspnoea at rest	1 (2,8)
Dyspnoea after moderate efforts	16 (44,4)
Asymptomatic	3 (8,3)

**HRCT pattern**	
UIP	13 (36,1)
cNSIP	17 (47,2)
fNSIP	6 (16,7)

**PFR**	
DLCO%	52 ± 16,1
FVC %	86,7 ± 26,4

BP: Blood pressure; PAPs: Mean pulmonary artery pressure estimation; UIP: usual interstitial pneumonia, cNSIP: cellular non-specific interstitial pneumonia; fNSIP: fibrotic non-specific interstitial pneumonia; DLCO: diffusing capacity of the lungs for carbon monoxide; FVC: Vital Forced Capacity.

Common comorbidities included hypertension (41,7%), hypercholesterolemia (16,7%), dysthyroidism (13,9%) and osteoporosis (11,1%). Two patients (5,5%) had recurrent pulmonary infections and one patient (2,8%) had diabetes mellitus. 22% of patients had previous or actual exposure to agents (tobacco use and history of environmental exposure ie, air pollution in 1 patient, domestic parrot exposure in 1 patient).

Most common respiratory symptoms at the onset of disease were shortness of breath with minimal-moderate exertion (44,4%), dry cough (44,4%), while a minority of patients (8,3%) was asymptomatic. In addition to ILD, arthritis was present at the onset in 16,6% of patients, a patient (2,8%) complained spreading myalgias, slight elevated CPK value (300 U/L) at laboratory analysis and EMG in normal range.

Several types of accompanying clinical manifestations were: Raynaud’s phenomenon in 9 patients (25%), fever in 5 patients (13,9%), puffy hands in 4 patients (11,1%), dry mouth in 4 patients (11,1%), dry eye in 3 patients (8,3%), telangiectasias in 2 patients (5,6%), dysphagia in 2 patients (5,6%). Only a patient (2,8%) presented Gottron papules and heliotrope rash, one patient presented calcinosis. No patient presented mechanic hands, Gottron sign, V sign, or distal ulcers.

Mean FVC was 86,7% ± 26,4 and the mean value of DLCO was 52% ± 16,1.

36,1% of patients demonstrated a UIP pattern by HRCT, 47,2% showed a cellular NSIP pattern and 16,7% showed a fibrotic NSIP pattern. Mean pulmonary arterial pressure (PAPs) estimation detected through echocardiography resulted elevated (≥ 25 mmHg) in 6 patients, among these patients the mean FVC was 99.6 ± 24 and mean DCLO 55 ± 25. For none, right heart catheterisation was indicated from cardiologist.

At the time of evaluation, half of the patients (50%) were taking steroids, hydroxychloroquine in two patients (5,5%), azathioprine in two cases (5,5%), mycophenolate mofetil in two cases (5,5%), pirfenidone in three cases (8,3%), nintedanib in one patient (2,7%).

As concerns serological findings, ANA ≥ 1/80 was the most common autoantibody (86,1%), followed by anti-Ro (38,9%), and anti-CCP (11,1%).

About ANA positivity, a centromeric pattern 1:80 and nucleolar pattern 1:160 were detected in two patients respectively. The other patients presented ANA≥1:320 with different fluorescence (**Table 3**).

**Table 3.** Serological features. Data are presented as n (%).

**ANA ≥ 1/80**	31 (86,1)
Homogeneous	5 (13,9)
Speckled	16 (44,4)
Centromere	3 (8,3)
Nucleolar	1 (2,8)
Cytoplasmic	6 (16,7)

**PL7**	3 (8,3)

**RO 52 kd**	8 (22,2)

**RO 60 kd**	6 (16,7)

**LA**	2 (5,5)

**PM-Scl**	2 (5,5)

**JO1**	3 (8,3)

**Ku**	2 (5,5)

**Sm-RNP**	2 (5,5)

**RNP**	1 (2,8)

**Scl-70**	1 (2,8)

**RNA Pol III**	1(2,8)

**FR**	2 (5,5)

**ACPA**	4 (11,1)

**ACA IgG/IgM, beta2GPI IgG/IgM**	1 (2,8)

**ds-DNA**	3 (8,3)

**U1 RNP**	1 (2,8)

**Nxp2**	1 (2,8)

**PCNA**	1 (2,8)

ANA ≥ 1/80, followed by anti-Ro, was the most frequent autoantibody in patients with UIP pattern and NSIP pattern at HRCT, ANA ≥ 1/80 and anti-Ro were the most frequent autoantibodies in patients with arthritis, while isolated positivity of anti-Ro was frequent in patients with Raynaud phenomenon. The frequency of the autoanti-bodies according to HRCT pattern and clinical features is also reported in **Table 4** and **5**.

**Table 4.** HRCT patterns and autoantibody profiles.

	**cNSIP**	**fNSIP**	**UIP**
**PL7**	3	0	0
**RO 52 kd**	4	2	2
**RO 60 kd**	3	1	2
**LA**	1	0	1
**PM-Scl**	0	1	1
**JO1**	1	1	1
**Ku**	1	0	1
**Sm-RNP**	0	1	1
**RNP**	0	1	0
**Scl-70**	1	0	0
**RNA Pol III**	0	1	0
**FR**	1	0	1
**ACPA**	1	0	3
**ACA IgG/IgM, beta2GPI IgG/IgM**	1	0	0
**ds-DNA**	3	0	0
**U1 RNP**	1	0	0
**Nxp2**	1	0	0
**ANA ≥ 1/80**	15	5	11

HRCT: High resolution CT; cNSIP: cellular non-specific interstitial pneumonia; fNSIP: fibrotic non-specific interstitial pneumonia; UIP: usual interstitial pneumonia.

**Table 5.** Clinical features and autoantibody profiles.

	**Raynaud p.**	**Puffy hands**	**Telangiectasias**	**Dry eye**	**Dry mouth**	**Arthritis**	**EF<60%**	**PAPs>25 mmHg**	**Dysphagia**
**PL7**	1	1	1	0	0	1	0	0	0
**RO 52 kd**	4	2	1	1	2	3	0	1	1
**RO 60 kd**	2	1	1	1	1	2	0	2	1
**LA**	0	0	0	1	1	1	0	1	0
**PM-Scl**	0	0	0	0	0	0	0	1	0
**JO1**	1	0	0	1	1	1	0	2	0
**Ku**	0	0	0	0	0	1	0	1	0
**Sm-RNP**	1	0	0	0	0	0	0	0	1
**RNP**	1	0	0	0	0	0	0	0	1
**Scl-70**	0	1	0	0	0	0	0	0	0
**RNA Pol III**	0	0	0	0	0	0	0	0	0
**FR**	0	0	0	1	1	1	0	0	0
**ACPA**	0	0	0	1	1	1	1	1	0
**ACA IgG/IgM, beta2GPI IgG/IgM**	0	0	0	0	0	0	0	0	0
**ds-DNA**	1	1	1	0	0	1	0	0	0
**U1 RNP**	0	0	0	0	0	1	0	0	0
**Nxp2**	0	0	0	0	0	1	0	0	0
**ANA ≥ 1/80**	5	4	2	2	4	5	1	6	2

EF: Ejection fraction; PAPs: Mean pulmonary artery pressure estimation.

NVC highlighted non-specific microangiopathy the most common pattern (50%), followed by non-specific pattern (38,9 %), scleroderma active pattern (8,3%) and normal pattern (2,8%).

NSIP pattern at HRTC was the most common pattern in patients with non-specific capillaroscopy meanwhile among UIP patients the most common pattern was non-specific microangiopathy (**Table 6**).

**Table 6.** HRCT patterns and Nailfold videocapillaroscopy patterns.

	**cNSIP**	**fNSIP**	**UIP**
**Normal**	1	0	0
**Non-specific**	8	2	4
**Non-specific microangiopathy**	8	3	7
**Early scleroderma pattern**	0	0	0
**Active scleroderma pattern**	0	1	2
**Late scleroderma pattern**	0	0	0

HRCT: High resolution CT; cNSIP: cellular non-specific interstitial pneumonia; fNSIP: fibrotic non-specific interstitial pneumonia; UIP: usual interstitial pneumonia.

Three patients presented a scleroderma pattern active at NVC, associated with ANA speckled fluorescence ≥1:320 in one patient; ANA cytoplasmatic pattern ≥1:1280 and RO52kd presence in two patients.

## DISCUSSION

In this study we described the clinical and serological characteristics of a cohort of IPAF patients from our centre. The mean age of our patients was 64,1± 9,3 years and this data is in line with most studies in literature.^7,8,9,10,11,12,13^

Few studies reported a younger mean age. ^14^

Predominant sex of our cohort was the female sex in agreement with other studies. ^7,8,10,11,13,14^ These characteristics of IPAF patients differ from those observed in CTD-ILD, where patients are predominantly female and younger. They are different from IPF patients too, who tend to be predominantly males and older.^7^ Conversely, in our cohort we reported a lower number of smokers than in most studies.^7,8,9,10,12,13,14^ Considering radiological patterns, NSIP was the predominant among our patients such as in other studies.^13,14^

UIP pattern at HRTC is considered to be a specific pattern of Rheumatoid Arthritis and it is not included in IPAF classification criteria; it is commonly reported in IPAF studies, and it was present in a high percentage of patients (36%) in our cohort. Only one study shows higher percentage.^8^

Such as in other cohorts, our IPAF group was more likely to present initially with respiratory manifestations, including cough and expectoration, dyspnoea, in comparison with CTD-ILD patients where other clinical manifestations are present at the onset.^15^ In our cohort of IPAF patients, Raynaud phenomenon was the most frequent clinical feature and was present in 25% of patients.

This data is in line with some studies from the literature.^8,11^

Moreover, some studies describe Raynaud phenomenon as the most frequent clinical feature, but with most consistent percentages of patients (39%–75%).^9,14^ Conversely, other clinical features described in most studies were not present in our cohort (mechanics hands) or were present with less frequency (arthritis).^7,9,12,13^ Moreover, we described other clinical features such as dry eyes and dry mouth (7 patients), dysphagia (2 patients), and skin involvement (heliotrope rash and calcinosis). These are all clinical aspects not included in IPAF classification criteria. A morbilliform and/or polymorphic rash of the face, neck, and extremities, was noted in approximately 54% of IPAF patients in a single-centre prospective study of Karampeli et al.^16^

In our cohort, a pulmonary hypertension (PH) was detected by echocardiogram in six patients. This result may have implication for management, including the need to detect PH at the time of the diagnosis of IPAF, and possibly to monitor for the risk of PH during follow-up of these patients.

Regarding serological domain, as in other studies, ANA was the most frequent marker in our patients, followed by anti-Ro and anti-CCP, while RF was less represented than in other cohorts (Oldam, Ito, Yoshimura, Kelly, Lim).^17^

Quantitative and qualitative nailfold features have been associated with pulmonary involvement in CTD but capillaroscopy studies in non-CTD-ILD populations, as IPF and IPAF patients, are limited.

In a study of Adelle S. Jee et al. CTD-ILD patients demonstrated lower mean capillary density, higher prevalence of giant capillaries, avascular areas and microhaemorrhages compared to IPAF and IPF patients, on the other hand nailfold characteristics didn’t differ between the latter two groups of patients.^18^

In our cohort the most frequent pattern was non-specific microangiopathy, characterized of decreased capillary density, more than 50% tortuous loops, enlarged and/or disarranged loops, more than 50% neoangiogenesis, and microhaemorrhages. We have reported among NSIP patients that the most frequent NVC pattern was non-specific meanwhile in UIP patients the most frequent was non-specific microangiopathy as reported in literature.^4^

Nintedanib is an antifibrotic drug that has demonstrated vascular remodelling in animal models.^19,20^ However, the impact of this drug on microvascular changes represented in NVC and whether this can be used to guide therapy remains to be determined. It could be interesting to follow up patients with nailfold scleroderma pattern and monitor patients undergoing nintedanib therapy.

We know from the literature that in CTD-ILD, a UIP pattern is generally associated with a better survival than UIP/IPF, ^21,22^ with the exception of RA-ILD patients with a UIP pattern who have an IPF-like survival.^23,24,25,26,27^

A possible future study could concern the follow-up of our cohort of IPAF patients and the evaluation of the prognosis of the UIP pattern in comparison with CTDILD/UIP patients from other cohorts.

IPAF patients may present with different clinical features. Some of them may present a myositis disorder while others may resemble more a systemic sclerosis. These clinical differences may have important implications on outcome.

So, attention should be put on clinical and immunologic features that would identify various subsets of IPAF that may have differential response to treatment.

Moreover, interstitial lung disease may be the initial manifestation of a CTD, and it is therefore possible for patients IPAF, to manifest a defined CTD at a later timepoint.^28,29^

In a study by Sebastiani et al.,^30^ evolution from IPAF to definite CTD was described in 13.5% of cases.

Another study by Park et al.^31^ showed that 10% of patients with an initial diagnosis of idiopathic NSIP developed a defined CTD at a median of almost 2 years after ILD diagnosis function tests.

## LIMITATIONS

Limitations of our study include the observational nature of the study, the lack of follow-up data and the relatively small sample size.

## CONCLUSION

In conclusion, we can underline the importance to study IPAF patients and follow them with the object to redefine IPAF classification criteria.

IPAF criteria could be redefined based on collected evidence according to data from retrospective and prospective studies recently published in literature.

## Abbreviations

ACA: Anti-cardiolipin antibodies
ACPA: Anti–citrullinated peptide antibodies
ANA: Antinuclear antibodies
Anti-dsDNA: Anti-double stranded DNA antibodies
ATS: American Thoracic Society
CBC: Complete Blood Count
CPK: Creatine PhosphoKinase
CRP: C-reactive protein
CTD: connective tissue disease
Complement: C3, C4
Cr: Serum Creatinine
DLCO: Diffusing Capacity of the Lungs for Carbon monoxide
ELISA: Enzyme Linked Immunosorbent Assay
EMG: Electromyography
ENA: Extractable nuclear antigen antibodies
ERS: European Respiratory Society
ESR: Erythrocyte Sedimentation Rate
FVC: Forced Vital Capacity
HRCT: High Resolution Computerised Tomography
IIFA: Indirect immunofluorescence assay
ILDs: Interstitial lung diseases
IPAF: Interstitial Pneumonia with Autoimmune Features
IPF: idiopathic pulmonary fibrosis
LDH: Lactic Dehydrogenase
LIP: Lymphocytic Interstitial Pneumonia
NSIP: Non-Specific Interstitial Pneumonia
NVC: Nailfold Video Capillaroscopy
OP: Organizing Pneumonia
PAPs: Pulmonary Arterial Pressure estimation
PFTs: Pulmonary Function Tests
PH: Pulmonary hypertension
RF: Rheumatoid Factor
SGOT: Serum Glutamic Oxaloacetic Transaminase
SGPT: Serum Glutamic Pyruvic Transaminase
SPEP: Serum Protein Electrophoresis
UIP: Usual Interstitial Pneumonia
bDMARDs: Biological Disease-Modifying Antirheumatic Drugs
beta2GPI: Anti-beta2-glycoprotein
cDMARDs: Conventional Disease-Modifying Antirheumatic Drugs
cNSIP: Cellular Non-Specific Interstitial Pneumonia
fNSIP: Fibrotic Non-Specific Interstitial Pneumonia
